# Women's preferences for HIV prevention service delivery in pharmacies during pregnancy in Western Kenya: a discrete choice experiment

**DOI:** 10.1002/jia2.26301

**Published:** 2024-07-05

**Authors:** Melissa Latigo Mugambi, Ben O. Odhiambo, Annabell Dollah, Mary M. Marwa, Judith Nyakina, John Kinuthia, Jared M. Baeten, Bryan J. Weiner, Grace John‐Stewart, Ruanne Vanessa Barnabas, Brett Hauber

**Affiliations:** ^1^ Department of Global Health University of Washington Seattle Washington USA; ^2^ Kenyatta National Hospital Nairobi Kenya; ^3^ UW‐Kenya Nairobi Kenya; ^4^ Department of Research and Programs Kenyatta National Hospital Nairobi Kenya; ^5^ Department of Medicine University of Washington Seattle Washington USA; ^6^ Department of Epidemiology University of Washington Seattle Washington USA; ^7^ Gilead Sciences Foster City California USA; ^8^ Department of Pediatrics University of Washington Seattle Washington USA; ^9^ Harvard Medical School and Division of Infectious Diseases Massachusetts General Hospital Boston Massachusetts USA; ^10^ The Comparative Health Outcomes Policy and Economics (CHOICE) Institute Department of Pharmacy University of Washington Seattle Washington USA; ^11^ Pfizer, Inc New York City New York USA

**Keywords:** Africa, differentiated care, HIV, HIV prevention, pregnant women, women

## Abstract

**Introduction:**

Pharmacy‐delivered HIV prevention services might create more options for pregnant women to use HIV prevention tools earlier and more consistently during pregnancy. We quantified preferences for attributes of potential HIV prevention services among women of childbearing age in Western Kenya.

**Methods:**

From June to November 2023, we administered a face‐to‐face discrete choice experiment survey to women aged 15–44 in Kenya's Homa Bay, Kisumu and Siaya counties. The survey evaluated preferences for HIV prevention services, described by seven attributes: service location, travel time, type of HIV test, sexually transmitted infection (STI) testing, partner HIV testing, pre‐exposure prophylaxis (PrEP) and service fee. Participants answered a series of 12‐choice questions. Each question asked them to select one of two service options or no services—an opt‐out option. We used hierarchical Bayesian modelling levels to estimate each attribute level's coefficient and understand how attributes influenced service choice.

**Results:**

Overall, 599 participants completed the survey, among whom the median age was 23 years (IQR: 18–27); 33% were married, 20% had a job and worked regularly, and 52% had been pregnant before. Participants, on average, strongly preferred having any HIV prevention service option over none (opt‐out preference weight: −5.84 [95% CI: −5.97, −5.72]). The most important attributes were the availability of PrEP (relative importance 27.04% [95% CI: 25.98%, 28.11%]), followed by STI testing (relative importance 20.26% [95% CI: 19.52%, 21.01%]) and partner HIV testing (relative importance: 16.35% [95% CI: 15.79%, 16.90%]). While, on average, participants preferred obtaining services at the clinic more than pharmacies, women prioritized the availability of PrEP, STI testing and partner HIV testing more than the location or cost.

**Conclusions:**

These findings suggest the importance of providing comprehensive HIV prevention services and ensuring PrEP, STI testing and partner HIV testing are available. If pharmacies can offer these services, women are likely to access those services at pharmacies even if they prefer clinics.

## 1 INTRODUCTION

Pharmacy‐delivered HIV prevention services are creating more options for care among priority populations globally [1, 2, 3]. These services could accelerate progress towards reducing HIV incidence, especially among women of childbearing age, who accounted for more than 50% of new HIV acquisitions in Eastern and Southern Africa between 2015 and 2019 [4].

However, the role of pharmacies in HIV prevention during pregnancy—a high‐risk period for HIV acquisition among women—is relatively unexplored. The World Health Organization and the Ministry of Health in Kenya recommend that antenatal care settings provide multiple options for HIV prevention among pregnant women, including risk reduction counselling, partner testing and linkage to care, screening, management of sexually transmitted infections (STIs) and pre‐exposure prophylaxis (PrEP) [5, 6]. While more than 90% of women are tested for HIV during pregnancy [7], the scale‐up of HIV prevention tools is still ongoing. As a result, the proportion of HIV‐negative women who use HIV prevention tools is uncertain and may be low [8]. Additionally, not all women access care early or regularly attend all antenatal care visits, limiting timely and continued access to HIV prevention [7].

Given that women of childbearing age frequently access pharmacies as an initial touchpoint for various reproductive services such as pregnancy testing [9, 10], pharmacies could be well‐positioned to address gaps in the antenatal care and HIV prevention continuum. However, no formal pharmacy models exist to optimize access to HIV prevention services among pregnant women globally. Furthermore, no studies have previously quantified women's preferences for receiving HIV prevention services during pregnancy globally. Pregnant women face unique demands during pregnancy [11], and understanding their preferences may influence how we design and implement HIV prevention services to ensure women initiate care early and remain engaged.

Implementation science increasingly recognizes the importance of deliberate and structured approaches to incorporating end‐user perspectives in service design before implementation to promote acceptability and adoption [12]. Discrete choice experiments (DCEs) offer a systematic methodology to design pharmacy‐based services by quantifying user preferences [13, 14]. DCEs simulate choice behaviour by asking people to choose among alternative services described by different characteristics or attributes [15, 16]. When people choose services in the survey, they make trade‐offs among attributes so that we can estimate the utility gained from improvements in each attribute and the relative importance of the attributes [17, 18]. We can use this information to decide which service components to prioritize, how much to charge and whom to engage [14, 19]. Such approaches to designing delivery services that incorporate what users value can offer opportunities to collaborate more effectively with the private sector, extend the reach of health services, and realize the impact of HIV prevention services [20].

In this study, we used a DCE to quantify preferences for delivering HIV prevention interventions (including PrEP, partner HIV testing and STI screening) among pregnant women. Our objective was to identify the most important attributes in choosing an HIV prevention service and the implications for service design.

## 2 METHODS

### 2.1 Survey development

We developed a DCE survey to explore women's preferences for HIV prevention services during pregnancy. The survey attributes were identified through a four‐step process. First, we identified 11 attributes informed by the research question, the literature and ongoing trials implementing PrEP in pharmacies [2, 3, 21, 22]. Second, we conducted seven focus group discussions with women (*n* = 52) to understand how they think about alternatives for getting HIV prevention services in pharmacies compared to what is currently available, determine what attributes and levels they care or do not care about and why and asked them to rank the attributes in order of importance. We purposefully recruited women of reproductive age from maternal and child health clinics in Homa Bay and Siaya and pharmacies in Kisumu. Women were eligible to participate if they had a pregnancy history or were considering becoming pregnant in the next 5 years.

Third, we conducted four focus group discussions with community health workers (*n* = 20) and nurses (*n* = 19) and eight individual key informant interviews to validate the findings and evaluate attribute feasibility, resulting in a preliminary list of seven attributes and levels. The technical experts (6 women, 2 men, ages 32–54) had training in nursing, pharmacy, medicine and social sciences, with experience in research and programme implementation related to HIV prevention and care for pregnant women. Finally, we designed and pre‐tested an initial survey instrument using think‐aloud cognitive interviews in English, Swahili or Dholuo to ensure we included relevant attributes critical to choosing an HIV prevention service [23].

The specific hypothetical service profiles and the profile pairings included in the DCE were determined by a D‐efficient experimental design that minimized D‐error, minimized overlapping attributes and excluded dominant pairs [15]. The experimental design was developed using Ngene (ChoiceMetrics, version 1.3.0, 2021) and comprised 72 hypothetical service pairs. To reduce the number of questions presented to each participant, we implemented a blocking strategy resulting in six blocks, each containing 12 questions. We randomized the order of the choice questions in each block 50 times, resulting in 300 survey versions programmed in Lighthouse Studio 9 (Sawtooth Software, version 9.15.0, Sawtooth Software LLC, Orem, UT). The survey included a description of the attributes and levels and two practice questions to prepare participants for the DCE. Questions outside of the DCE explored potential influences on women's preferences, such as their experiences with pharmacies, antenatal care and HIV prevention services, their attitudes towards pharmacy and clinic services, socio‐demographic details, expenditure on pharmacies and antenatal care clinics, and participants’ perceptions of the difficulty of answering the DCE questions.

We conducted a pilot test of the survey instrument with 42 women to evaluate the clarity and comprehension of the choice tasks and general survey questions, how the attributes and levels affected participants’ preferences, translation accuracy, survey performance on the Sawtooth platform and ease of navigation. In the initial version of the survey instrument, participants chose the highest service fee level (750 KSH) 48% of the time, indicating that this service fee was unlikely to approach the maximum service fee participants would be willing to pay for HIV prevention services. Research suggests that the maximum level of price, cost or fee should not be chosen by participants more than approximately 25% of the time if the disutility of price increases is to be used in estimating willingness to pay [24]. Therefore, we increased the service fee range levels to 0 KSH, 400 KSH and 1000 KSH. We also made minor wording and structural edits to improve participants’ understanding of the questions. The final survey consisted of a series of choice questions where participants were asked to choose among three alternatives: two hypothetical service profiles provided by a trained health provider and no HIV prevention services in pregnancy. Each hypothetical service was described by seven attributes (Table 1).

**Table 1:** Attribute descriptions and levels used to define the service alternatives in each choice question

Attribute	Levels
The location where the services are provided	You go to a clinic for pregnant women You go to a pharmacy with a private room You go to a pharmacy without a private room
How long it takes to travel to the location	It takes you 15 minutes to get there It takes you 30 minutes to get there It takes you 60 minutes to get there
The type of HIV test you use	You test yourself with a saliva test You test yourself with a blood‐based finger prick test A provider tests you with a blood‐based finger prick test
Whether you can take an HIV test to your partner^1^	You cannot take an HIV test to your partner You can take an HIV test to your partner
Whether STI testing is available at the location^2^	STI testing is not available STI testing is available
Whether PrEP pills are available	PrEP is not available PrEP is available
How much you pay for the services	You do not pay anything for the services You pay 400 KES for the services You pay 1000 KES for the services

*Note*: Additional information provided to participants: 1. Before you take a test to your partner, the provider will give you instructions on conducting the test. 2. Additional information provided to participants: You can catch sexually transmitted infections (STIs), such as syphilis, gonorrhoea and chlamydia, during sex. STI testing would involve providing a urine sample for gonorrhoea and chlamydia testing and having the provider conduct a blood‐based finger prick test for syphilis. Any STIs found will be treated.

Abbreviations: KES, Kenyan shilling; PrEP, pre‐exposure prophylaxis.

### 2.2 Study setting and recruitment

From June to November 2023, we administered a DCE survey in three counties in Western Kenya: Kisumu, Homa Bay and Siaya. More details about the study setting have been described elsewhere [25]. Recruitment occurred in four phases, ensuring representation across counties for different groups defined by age (15–17, 18–24, 25–44), prior pregnancy (previous pregnancy vs. never pregnant) and employment status (employed vs. not employed) that might influence participant preferences. First, we engaged participants from prior studies implementing PrEP in maternal and child health clinics and pharmacies [3, 22, 26, 27]. Second, we engaged participants from public health facilities (two each in Homa Bay and Siaya) and pharmacies (13 in Kisumu) participating in prior and ongoing studies [28]. Facilities were selected based on logistical feasibility and willingness to participate. Third, we recruited participants from pharmacies within the facility catchment areas (Homa Bay and Siaya) and continued recruiting participants from Kisumu pharmacies. We collaborated with pharmacy associations to identify legally registered pharmacies in Homa Bay (*n* = 7) and Siaya (*n* = 6), located in urban and rural areas, offering sexual and reproductive health services, accessible to research assistants and willing to participate. Finally, we focused on recruiting adolescent participants aged 15–17.

Six trained research assistants (two in each county) used multiple recruiting strategies depending on the recruitment phase, including contacting a randomized list of pre‐screened prior participants via phone, liaising with staff (e.g. clinic and pharmacy providers, community health workers and study coordinators) to identify potentially eligible participants, publicizing the study at facility sites, identifying walk‐ins at pharmacies and snowballing—particularly in the case of adolescents. At facility sites, we focused on multiple types of clinics for recruitment, including family planning and maternal and child health clinics. To assess potential participants’ eligibility, research assistants administered a screening survey using Sawtooth software (version 9.15.0; Sawtooth Software LLC) in private facility areas or study offices. Women were eligible to participate if they self‐reported being HIV negative, were aged 15–44, had ever been pregnant or intended to become pregnant within the next 5 years and were willing to join the study. Women under 18 were only eligible if they had experienced pregnancy, given their status as emancipated minors.

### 2.3 Data collection and analysis

Once participants were screened and consented to participate, research assistants arranged for them to take the survey in a private facility area or the study office. The research assistants used tablets with the Sawtooth app to conduct the survey in person. This allowed participants to view the questions on the screen while responding.

We analysed participants’ key characteristics, experiences and attitudes using descriptive statistics. Because we revised the levels and expanded the range of the service fee levels in the DCE after the pilot, we tested whether the differences in the levels and range of levels of this attribute would impact the results of the analysis. To test this, we created an interaction term by multiplying the service fee by a dummy variable equal to 1 if the data were from the pilot. We included this term in a conditional logit model, where the service fee was modelled as a continuous variable. Because the interaction term was not statistically significant, we pooled data from the pilot phase and main survey. We used a hierarchical Bayesian (HB) model to estimate each attribute level coefficient (or preference weight) and understand how each attribute influenced each participant's choices of service profiles. HB models first estimate a unique set of preference weights for each respondent in an effects‐coded conditional logit model. The model then uses individual preference weights to construct a joint posterior distribution of preference weights for the total sample, including the average and standard deviation [17]. We then estimated the average relative importance of each attribute across the entire sample by averaging the individual relative importance values [17]. In subgroup analyses, we evaluated how relative importance varied among participants with different characteristics, including county, age, employment status and parity. Statistical analyses were conducted using STATA (version 17.0; StataCorp LP, College Station, TX) and Sawtooth software (version 9.15.0; Sawtooth Software LLC).

### 2.4 Ethical approval

The study was approved by the Kenyatta National Hospital—University of Nairobi Ethical Research Committee, the National Commission for Science, Technology, and Innovation, and the University of Washington Institutional Review Board. All participants provided written informed consent before participating in the study.

## 3 RESULTS

Table 2 summarizes the demographic and behavioural characteristics of survey participants. We screened 645 women for eligibility and enrolled 600 (93%) women who met the eligibility criteria. Data from 599 women were included in the analysis. Participants had a median age of 23 years (IQR: 17–27) and came from three counties: Kisumu (33%), Homa Bay (33%) and Siaya (33%). One hundred and seven (18%) participants had participated in prior studies. Thirty‐three percent of participants were married, 20% had a job and worked regularly, 18% had non‐regular or consistent work and 43% had completed at least some secondary school education. Sixty‐three percent visited a pharmacy once a month or more in the 12 months preceding the survey. Three hundred and eleven women (52%) reported being pregnant before, with a median number of 1 child (IQR: 1–2). When reflecting on their experience of service use during their most recent pregnancy, 98% had visited an antenatal care clinic, 98% had received an HIV test, 56% had their partners tested for HIV, 68% had received STI testing and 13% had taken PrEP. Among all women surveyed about their experience of service use when not pregnant (*n* = 599), 87% reported testing for HIV, 60% had their partners tested for HIV, 35% received STI testing and 15% took PrEP.

**Table 2:** Participant demographic and behavioural characteristics

		Total (*N* = 599)
Age (y)	Median (interquartile range)	23 (17, 27)
County location	Kisumu	200 (33%)
	Homa Bay	199 (33%)
	Siaya	200 (33%)
Recruitment site	Prior studies	107 (18%)
	Health facilities	193 (32%)
	Pharmacies	299 (50%)
Relationship status	Married	200 (33%)
	Single	200 (33%)
	Divorced/separated	7 (1%)
	In a relationship	179 (30%)
	Living with a partner	13 (2%)
Education completed	At least some primary school	197 (33%)
	At least some secondary school	255 (43%)
	At least some higher education	139 (23%)
	Never attended school	8 (1%)
Employment status	Has a job and works regularly	118 (20%)
	Has work, but it is not regular or consistent	106 (18%)
	Currently does not have a job	229 (38%)
	Currently in school and not working	146 (24%)
Pharmacy visits in the past 12 months	Once a month or more	376 (63%)
	Every 2 or 3 months	133 (22%)
	1 or 2 times a year	83 (14%)
	Not at all	7 (1%)
Travel time to regularly visited pharmacy	Less than 15 minutes	311 (52%)
	15–29 minutes	202 (34%)
	30–59 minutes	76 (13%)
	≥ 60 minutes	10 (2%)
Prior pregnancy	Yes	311 (52%)
	No	288 (48%)
Number of children^a^	Median (interquartile range)	1 (1, 2)
Ever visited a clinic for pregnant women^a^	Yes	304 (98%)
	No	7 (2%)
Conducted HIV test when pregnant^a^	Yes	305 (98%)
	No	6 (2%)
Partner tested for HIV when pregnant^a^	Yes	175 (56%)
	No	115 (37%)
	Not sure	21 (7%)
Conducted STI test when pregnant^a^	Yes	213 (68%)
	No	92 (30%)
	Not sure	6 (2%)
Took PrEP when pregnant^a^	Yes	39 (13%)
	No	272 (87%)
Conducted HIV test when *not* pregnant	Yes	520 (87%)
	No	79 (13%)
Partner tested for HIV when *not* pregnant	Yes	358 (60%)
	No	176 (29%)
	Not sure	65 (11%)
Conducted STI test when *not* pregnant	Yes	208 (35%)
	No	390 (65%)
	Not sure	1 (0.2%)
Took PrEP when *not* pregnant	Yes	88 (15%)
	No	511 (85%)

Abbreviations: PrEP, pre‐exposure prophylaxis; STI, sexually transmitted infection.

^a^ Data shown reflect responses from those with prior pregnancies (*n* = 311).

Fifty‐four percent of participants completed the choice questions primarily in English, 25% in Dholuo and 22% in Swahili. On a 5‐point verbal rating scale (very difficult, difficult, normal, easy, very easy), 88% of the participants found the survey normal or easy to complete. Participants completed all choice tasks. One participant always selected “service A” or “service B” across all choice questions, potentially indicating that the participant was not paying attention to the choice questions and, therefore, was removed from the analysis. Across choice questions, 91% of participants always chose a service option, 9% sometimes opted out and no participants always opted out.

### 3.1 Preference results

Table 3 shows the mean preference weights, standard deviations and 95% confidence intervals (CIs) for the attribute levels. Higher mean preference weights suggest greater preference on average for the select attribute level. All attributes demonstrated significance and were important in choosing an HIV prevention service. Participants, on average, preferred having any HIV prevention service option rather than none, as reflected by the large negative preference weight on the no‐service alternative (−5.84 [95% CI: −5.97, −5.72]). Overall, women most preferred going to a clinic to receive HIV prevention services (compared to a pharmacy with a private room and a pharmacy without a private room), taking 15 minutes to travel to the location (compared to 30 or 60 minutes), provider‐assisted HIV testing (compared blood‐based or saliva‐based HIV self‐tests), the availability of PrEP, STI testing and partner testing (compared with no availability) and not paying anything for the service (compared to 400 Kenyan shillings which in turn was preferred to 1000 Kenyan shillings).

**Table 3:** Attribute‐level preference weights (*N* = 599)

Label	Utility	Std deviation	Lower 95% CI	Upper 95% CI
None	−5.84	1.54	−5.97	−5.72
Location				
Clinic	0.34	0.35	0.31	0.36
Private pharmacy	0.09	0.42	0.06	0.13
Pharmacy	−0.43	0.49	−0.47	−0.39
Travel time				
15 minutes	0.17	0.38	0.14	0.20
30 minutes	−0.05	0.27	−0.07	−0.03
60 minutes	−0.11	0.27	−0.14	−0.09
HIV test type				
Saliva test	−0.09	0.32	−0.11	−0.06
Blood test	−0.14	0.29	−0.16	−0.11
Provider test	0.23	0.36	0.20	0.25
STI testing				
STI not available	−1.19	0.66	−1.24	−1.14
STI available	1.19	0.66	1.14	1.24
Partner HIV testing				
Partner test not available	−0.94	0.57	−0.98	−0.89
Partner test available	0.94	0.57	0.89	0.98
PrEP availability				
PrEP not available	−1.59	0.92	−1.66	−1.51
PrEP available	1.59	0.92	1.51	1.66
Service fee				
Free	0.51	0.56	0.47	0.56
400 KSE	0.11	0.31	0.09	0.14
1000 KSE	−0.62	0.58	−0.67	−0.58

*Note*: Utilities represent relative preferences for each level within each attribute. Higher values indicate a stronger preference.

Abbreviations: CI, confidence interval; KSE, Kenyan Shilling; PrEP, pre‐exposure prophylaxis; STI, sexually transmitted infection.

Although women preferred the clinic on average, the individual utility estimates obtained from the HB analysis revealed that 36% (*n* = 215) of women preferred a pharmacy with a private room over a clinic, while 13% (*n* = 77) preferred a pharmacy without a private room over a clinic (data not shown). Changing the location from a pharmacy without a private room to a clinic was three times as important as changing the location from a pharmacy with a private room to a clinic. In a question where participants ranked the privacy of a clinic, pharmacy with a private room and pharmacy without a private room, with 1 being the setting where they felt the most privacy and 3 being the setting where they felt the least privacy, 56% (*n* = 339) ranked a pharmacy with a private room as the most private, 42% (*n* = 252) ranked a clinic as the most private and 2% (*n* = 10) ranked a pharmacy without a private room as the most private.

**Figure 1:**
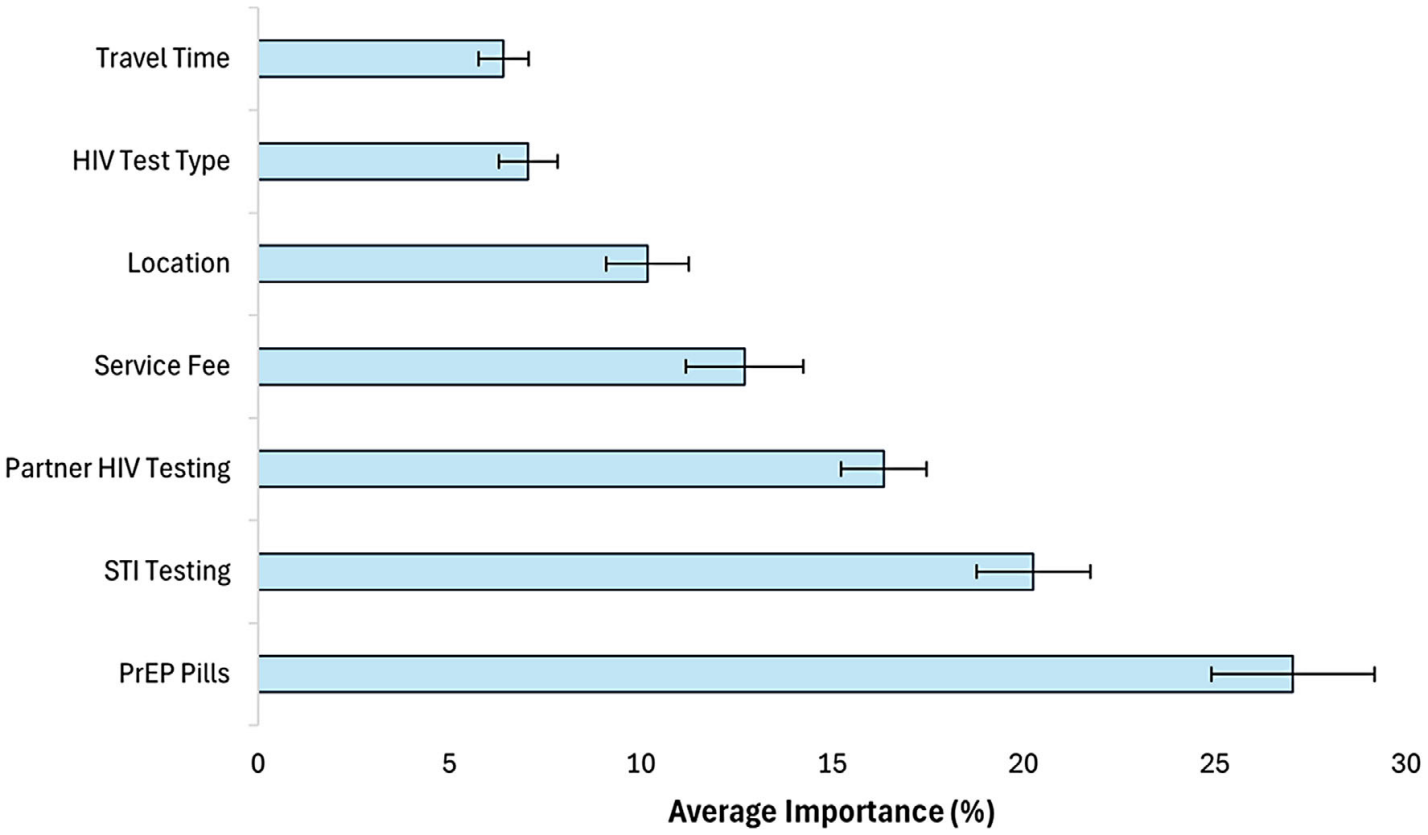
**Average relative importance of the attributes and 95% confidence intervals**. Average relative importance is calculated by taking the difference in preference weights between each attribute's most preferred and least preferred level, dividing this difference by the sum of the differences across all attributes, and multiplying by 100. The attribute importance values add up to 100%. Data are ratio‐scaled; therefore, an attribute with an importance score of 20% is twice as important as an attribute with a score of 10%. *N* = 599. Abbreviations: PrEP, pre‐exposure prophylaxis; STI, sexually transmitted infection.

Figure 1 shows each attribute's average relative importance scores (and 95% confidence interval). Among the seven attributes evaluated, PrEP availability was the most important determinant of service choice, accounting for 27.04% (95% CI: 25.98%–28.11%) of the impact on treatment choice, followed by STI testing availability (20.26% [95% CI: 19.52%–21.01%]), partner HIV testing availability (16.35% [95% CI: 15.79%–16.90%]), service fee (12.71% [95% CI: 11.94%–13.48%]), location (10.17% [95% CI: 9.63%–10.71%]), HIV test type (7.06% [95% CI: 6.68%–7.44%]) and travel time (6.41% [95% CI: 6.09%–6.74%]). The relative importance significantly differed among all attributes except between HIV test type and travel time. The mean relative importance scores stratified by age, employment status, prior pregnancy and county location were qualitatively consistent with and generally had the same general rank order as those for the entire sample—PrEP, STI testing and partner testing consistently accounted for the greatest proportion of the decision‐making importance (data not shown).

## 4 DISCUSSION

We conducted a DCE survey to evaluate the preferences of women of reproductive age in Western Kenya for HIV prevention service delivery during pregnancy. We found that all else equal, women placed a higher value on the availability of PrEP, STI testing and partner HIV testing compared to the logistical considerations of where the service would be provided or how much it would cost. Service fee and location were each less critical but together had a relative importance of 23%. On average, most women preferred free or lower‐cost options and accessing care at a clinic versus a pharmacy.

Several factors might have contributed to women's preference for clinics over pharmacies, including the perceived availability of well‐trained providers, low‐cost comprehensive services, as well as ongoing efforts by the Ministry of Health to encourage antenatal care clinic utilization [29, 30, 31, 32]. Promoting pharmacy use for HIV prevention among pregnant women is a new concept supported by recent studies on pharmacy‐delivered PrEP and private‐sector engagement in primary care [3, 26]. This might explain why over a third of women in our study preferred pharmacies with private rooms over clinics. Our results also indicate that more women felt that a pharmacy with a private room offered more privacy than a clinic; therefore, providing a private room in a pharmacy might encourage women to use such services [33]. However, to make pharmacies more acceptable, other improvements are needed, such as strengthening and enhancing the perception of providers’ skillsets [33, 34].

Interestingly, despite most participants not using PrEP, its availability was the most important attribute, suggesting high awareness and perceived importance among women of reproductive age in Western Kenya [35, 36]. While our formative work highlights the importance of chlamydia and gonorrhoea testing for women, particularly in the context of protecting their infants during pregnancy, limited literature exists on the awareness of the importance of testing more broadly in Western Kenya. A study in another region in Kenya found low awareness of the importance of STI testing, especially for asymptomatic individuals, suggesting that prevention campaigns have had more emphasis on reducing the risk of HIV or syphilis compared to chlamydia or gonorrhoea [37]. A previous study implementing PrEP in Western Kenyan maternal and child health clinics from 2018 to 2019 found a higher uptake of HIV self‐tests for partner testing compared to PrEP [38]. PrEP, however, offers women the potential benefit of personal control over HIV prevention, independent of their partners [27, 39]. Further qualitative research would be beneficial to understand these findings.

These findings suggest the importance of providing comprehensive HIV prevention services and ensuring that PrEP, STI testing and partner testing are a core part of any HIV prevention service. Community‐based approaches can ensure women access prevention tools early and consistently during pregnancy [40], and pharmacies could play a pivotal role in bridging these gaps while being of value to women. Given the promising acceptability and utilization of pharmacy‐delivered PrEP among adolescent girls and young women, it is essential to ensure equitable access to pharmacy‐based services for pregnant women [11, 26]. As these models are tested and refined, we need to define the specifics of integrating care within the broader antenatal care system and how to improve pharmacy‐based models to create more tailored care options.

In this study, the cost of services was not one of the more important attributes, which differs from other studies evaluating preferences for HIV products or services [41, 42]. Our cost range may have been too low to capture participants’ willingness to pay, making the service fee less influential. Typically, the highest cost level should approach the highest amount a participant would be willing to pay [24]. While our study was not designed to estimate willingness to pay, it is important to recognize that some participants may be willing to pay more than 1000 Kenyan shillings (∼ 7 USD as of 2023 when the study was conducted) for the service. A 2023 Kenyan study investigating preferences for online PrEP delivery found that 50% and 25% of adults were willing to pay up to 1000 and 1975 Kenyan shillings, respectively [43]. Future research should explore the willingness to pay for pharmacy‐based HIV prevention services during pregnancy, pharmacy delivery costs and their impact on service fees, and the potential of cost‐sharing strategies for implementation.

We acknowledge that this study recruited participants from health facilities and pharmacies in Western Kenya (with 18% of participants having prior PrEP experience), which may limit generalizability and potentially influence preferences due to familiarity with HIV prevention services in the region [35, 44]. We also acknowledge the inherent limitation of DCE surveys, where it is not always immediately apparent to participants what they care about in theory until they use the service [45, 46]. Notably, this DCE included attributes with varying levels. Three of these attributes had two levels, representing the availability or non‐availability of HIV prevention services. These attributes are likely to be more salient to participants, as the inherent preference for having a service available versus not exerts a stronger influence on choices [47]. However, our objective was to determine whether participants cared about including these services in a prevention package. Further research is needed to identify the specific components of each service and explore how they can be effectively operationalized in a pharmacy setting that aligns with the needs of women of reproductive age, providers and policymakers.

## 5 CONCLUSIONS

In conclusion, this study in Western Kenya evaluated the preferences for HIV prevention service delivery during pregnancy among women in three counties. The study found that, on average, women strongly preferred any HIV prevention service over no service at all. This demonstrates a need for continued efforts to increase the uptake of prioritized services (PrEP, STI testing and partner HIV testing) and explore diverse delivery channels, like pharmacies, where women would be willing to access such services if available. Interestingly, participants were less sensitive to the cost of these services, meaning they were willing to pay more than the highest amount the study offered. Further research is needed to determine whether these results would hold for other groups of women, understand the contexts in which pharmacy‐based delivery makes sense and for whom, and how to best integrate these options within the broader provision of antenatal care services.

## COMPETING INTERESTS

MLM reports grants from the National Institutes of Health (NIH). RVB reports grants from King K. Holmes Professorship in STDs and AIDS, Bill and Melinda Gates Foundation (BMGF), National Institutes of Health (NIH), and manuscript and abstract writing support from Regeneron Pharmaceuticals outside the submitted work. BH reports stock options from Pfizer and grants from NIH outside the submitted work. BH is a paid employee at Pfizer. JMB is a paid employee at Gilead Sciences. GJ‐S reports grants from NIH, CDC, Thrasher Foundation, IMPAACT and stock options from Malaika outside the submitted work. GJ‐S sits on the DSMB of the VITALITY and Tatelo trials.

## AUTHORS’ CONTRIBUTIONS

MLM and BH designed the study; MLM acquired funding; BOO, AD and JN managed the study site and oversaw recruitment and pharmacy engagement efforts; MMM and MLM analysed the data; GJ‐S, BJW, JMB, JK and RVB provided expertise in HIV prevention and pharmacy‐based delivery, and MLM wrote the initial manuscript. All authors contributed to and approved the final manuscript.

## FUNDING

A National Institutes of Mental Health Grant K01MH122326 provided financial support for this study.

## CME STATEMENT

This article is published as part of a supplement supported by unrestricted educational grant by ViiV Healthcare.

Credits Available for this Activity: American Medical Association (AMA Credit).

Washington University School of Medicine in St. Louis designates this enduring material for a maximum of 1 AMA PRA Category 1 Credit™. Physicians should claim only the credit commensurate with the extent of their participation in the activity.

## Data Availability

The data supporting this study's findings are available from the corresponding author upon reasonable request.
